# Development and evaluation of an up-converting phosphor technology-based lateral flow assay for rapid and quantitative detection of *Coxiella burnetii* phase I strains

**DOI:** 10.1186/s12866-020-01934-0

**Published:** 2020-08-12

**Authors:** Pingping Zhang, Jun Jiao, Yong Zhao, Mengjiao Fu, Jin Wang, Yajun Song, Dongsheng Zhou, Yongqiang Wang, Bohai Wen, Ruifu Yang, Xiaolu Xiong

**Affiliations:** 1 State Key Laboratory of Pathogen and Biosecurity, Beijing Institute of Microbiology and Epidemiology, Beijing, P. R. China; 2 Beijing Key Laboratory of POCT for Bio-emergency and Clinic (No.BZ0329), Beijing, P. R. China; 3 Preventive Veterinary Medicine, College of Veterinary Medicine, China Agricultural University, Beijing, P. R. China

**Keywords:** *Coxiella burnetii*, Q fever, Lipopolysaccharide, Up-converting phosphor technology-based lateral flow, Monoclonal antibody

## Abstract

**Background:**

*Coxiella burnetii* is an obligate intracellular Gram-negative bacterium that causes a zoonotic disease commonly called Q fever globally. In this study, an up-converting phosphor technology-based lateral flow (UPT-LF) assay was established for the rapid and specific detection of phase I strains of *C. burnetii*.

**Results:**

Specific monoclonal antibodies (10B5 and 10G7) against *C. burnetii* phase I strains were prepared and selected for use in the UPT-LF assay by the double-antibody-sandwich method. The detection sensitivity of the *Coxiella*-UPT-LF was 5 × 10^4^ GE/ml for a purified *C. burnetii* phase I strain and 10 ng/ml for LPS of *C. burnetii* Nine Mile phase I (NMI). Good linearity was observed for *C. burnetii* phase I and NMI LPS quantification (R^2^ ≥ 0.989). The UPT-LF assay also exhibited a high specificity to *C. burnetii*, without false-positive results even at 10^8^ GE/ml of non-specific bacteria, and good inclusivity for detecting different phase I strains of *C. burnetii*. Moreover, the performance of the *Coxiella*-UPT-LF assay was further confirmed using experimentally and naturally infected samples.

**Conclusions:**

Our results indicate that *Coxiella*-UPT-LF is a sensitive and reliable method for rapid screening of *C. burnetii,* suitable for on-site detection in the field.

## Background

*Coxiella burnetii* is an intracellular Gram-negative bacterium that causes a zoonotic disease known as Q fever globally. It can undergo a phase transition that is correlated with some of the biological characteristics of the “smooth-to-rough” lipopolysaccharide (LPS) variation observed for Gram-negative Enterobacteriaceae [1]. The virulent form of *C. burnetii* (phase I, PI) has full-length LPS and is usually isolated from natural and laboratory infections [2]. Upon serially passaging in embryonic cells, tissue culture, or synthetic medium, a smooth-to-rough (truncated) LPS transition occurs, which results in avirulence (phase II, PII) [3]. *C. burnetii* PI is able to replicate in immunocompetent hosts and is considered as a category B bio-warfare agent [4], which makes it a public health and biosecurity concern.

Diagnosis of Q fever is difficult due to the lack of distinct clinical features that distinguish it from other febrile diseases [5]. Currently, the diagnosis of Q fever mainly depends on the detection of antibodies or nucleic acids. Serological methods, especially immunofluorescence assays, are considered as the reference methods for diagnosis of Q fever in both humans and animals. However, an important drawback to serological diagnosis of acute Q fever is the lag phase in antibody response of 7–15 days after onset of clinical symptoms, limiting early diagnosis [6]. Although PCR-based approaches provide a more sensitive way for the detection of Q fever, the results of PCR testing of peripheral blood are variable and do not provide information on the viability of *C. burnetii* [7, 8]. Moreover, the need for expensive equipment and professional training is also a barrier for the use of these methods in primary laboratories and in the field.

To prevent or minimise Q fever outbreaks in humans, rapid, simple, sensitive, and accurate methods for *C. burnetii* detection in natural infections and for potential bioterrorist attacks are still needed. Recently, an up-converting phosphor technology-based lateral flow (UPT-LF) assay using up-converting phosphor particles (UCPs) as the bio-label, with excitation and emission peaks at 980 and 541.5 nm, has been developed as a new point-of-care testing method. UPT-LF exhibits high sensitivity and stability, as well as robust performance when tested with complex samples [9–12]. In the current study, a UPT-LF assay for the rapid and specific detection of PI strains of *C. burnetii* was established. The performance of this assay was comprehensively evaluated with cultured material and experimentally and naturally infected samples.

## Results

### Development of *Coxiella*-UPT-LF

The monoclonal antibodies (mAbs) against *C. burnetii* were prepared in mice that were immunised with purified *C. burnetii* Xinqiao strain (PI). Three cloned hybridomas (10B5, 10G7, and 13D6) that produced *C. burnetii* PI-specific mAb and two cloned hybridomas (6D8 and 8A1) that produced both PI- and PII-specific mAb were identified by ELISA analysis of hybridoma supernatants with PI and PII antigens. The isotypes, concentrations, and potencies of these mAbs are listed in Table 1.

**Table 1:** The monoclonal antibodies against *C. burnetii*

Antibody	Isotype	Potency		Concentration (mg/ml)
		PI-specific mAb (PI Xinqiao strain)	PII-specific mAb (PII Grita strain)	
10B5	IgM	62.5 ng/ml	–	8
10G7	IgM	31.2 ng/ml	–	5
13D6	IgG1	15.6 ng/ml	–	10
6D8	IgM	125 ng/ml	125 ng/ml	6
8A1	IgG2b	62.5 ng/ml	7.8 ng/ml	14

**Fig. 1:**
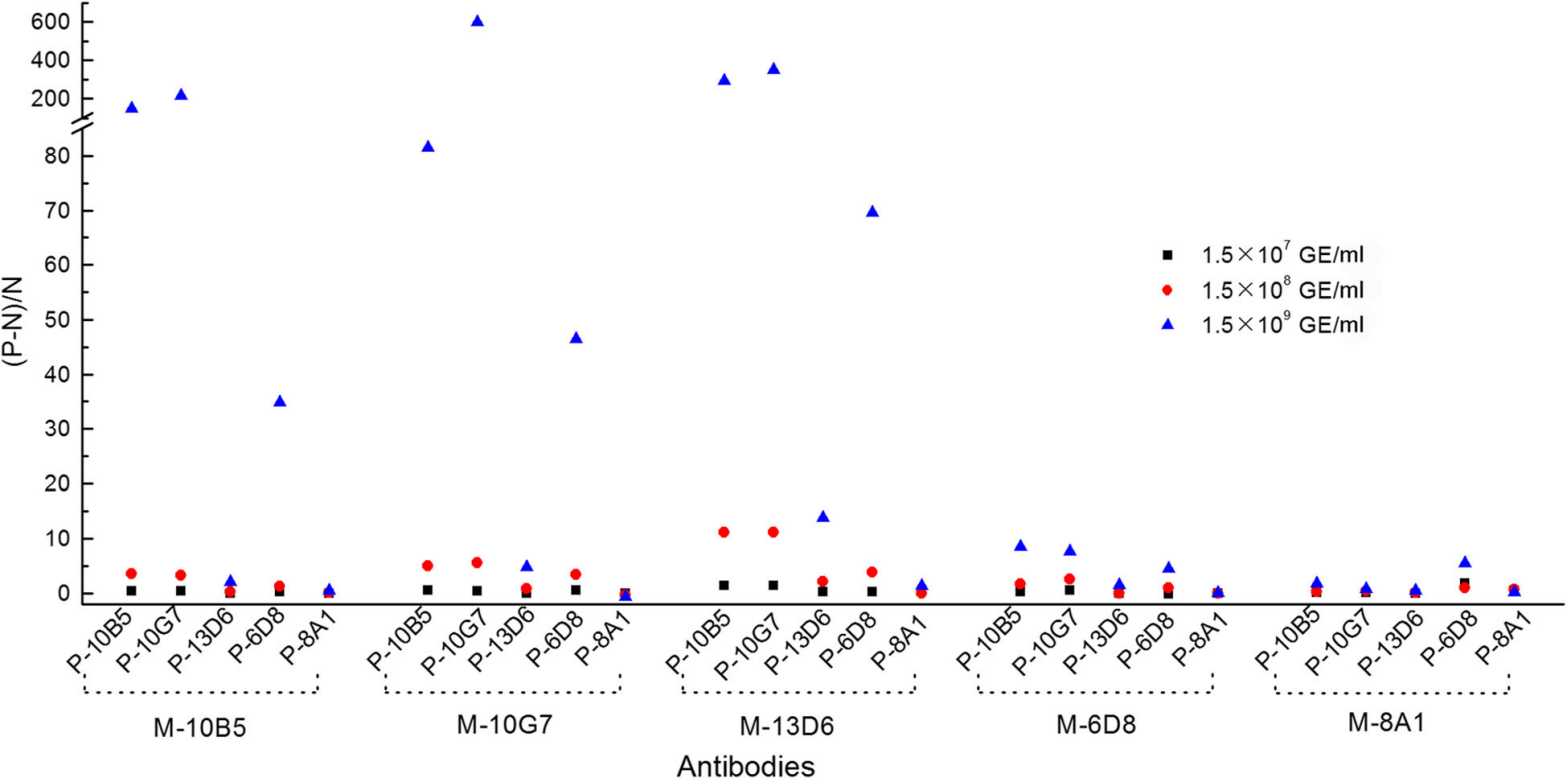
Assessment of UPT-LF strips fabricated using different antibodies. Nitrocellulose membranes and conjugation pads both with various antibodies were paired randomly in the fabrications of the strips, and each strip was used for the detection of *C. burnetii* Xinqiao strain at three different concentrations with primary sample treating buffer and different labelling conditions between UCPs and antibodies. The letter “P” before the name of the antibodies means that the antibodies were on the conjugate pad, while the letter “M” means they were on the nitrocellulose membrane

To screen for suitable antibodies for the UPT-LF assay, strips fabricated with conjugation pads and nitrocellulose membranes with various antibodies were evaluated using a *C. burnetii* Xinqiao strain purified from yolk sac (YS) with primary sample treating buffer and different labelling conditions between UCPs and antibodies. The results are shown in Fig. 1. Strips with nitrocellulose membrane with 10B5, 10G7, and 13D6 paired with conjugation pads with 10G7 or 10B5 showed excellent performances. After further optimisation of the sample treating buffer components, as well as optimisation of labelling conditions between UCP and antibodies, *Coxiella*-UPT-LF strips with 10G7 on the nitrocellulose membrane and 10B5 on the conjugation pad were finally selected; the corresponding sample treating buffer was 0.03 M phosphate buffer containing 0.5% IGEPAL CA-630 and 0.1 M NaCl.

The amino acid sequences of variable heavy (VH) and variable light (VL) chains of 10B5 and 10G7 were determined by cDNA sequencing. The sequences of the VH and VL chains of 10B5 and 10G7 were identical, indicating that they were subclones from the same fusion event (Fig. S1).

### Detection limit, sensitivity, and precision

Samples with purified *C. burnetii* Xinqiao strain at concentrations from 1 × 10^3^ to 1 × 10^8^ genome equivalents (GE)/ml or Nine Mile PI (NMI) or PII (NMII) LPS from 1 to 10,000 ng/ml were analysed in triplicate. Phosphate buffer was measured 10 times, and the mean plus three standard deviations was set as the cut-off value, which was 0.097. Samples with a T/C ratio higher than the cut-off value were considered as positive. The lowest concentration with at least one positive result higher than the cut-off was defined as the detection limit of *Coxiella*-UPT-LF; the lowest concentration with three positive results out of three tests was defined as the sensitivity.

**Fig. 2:**
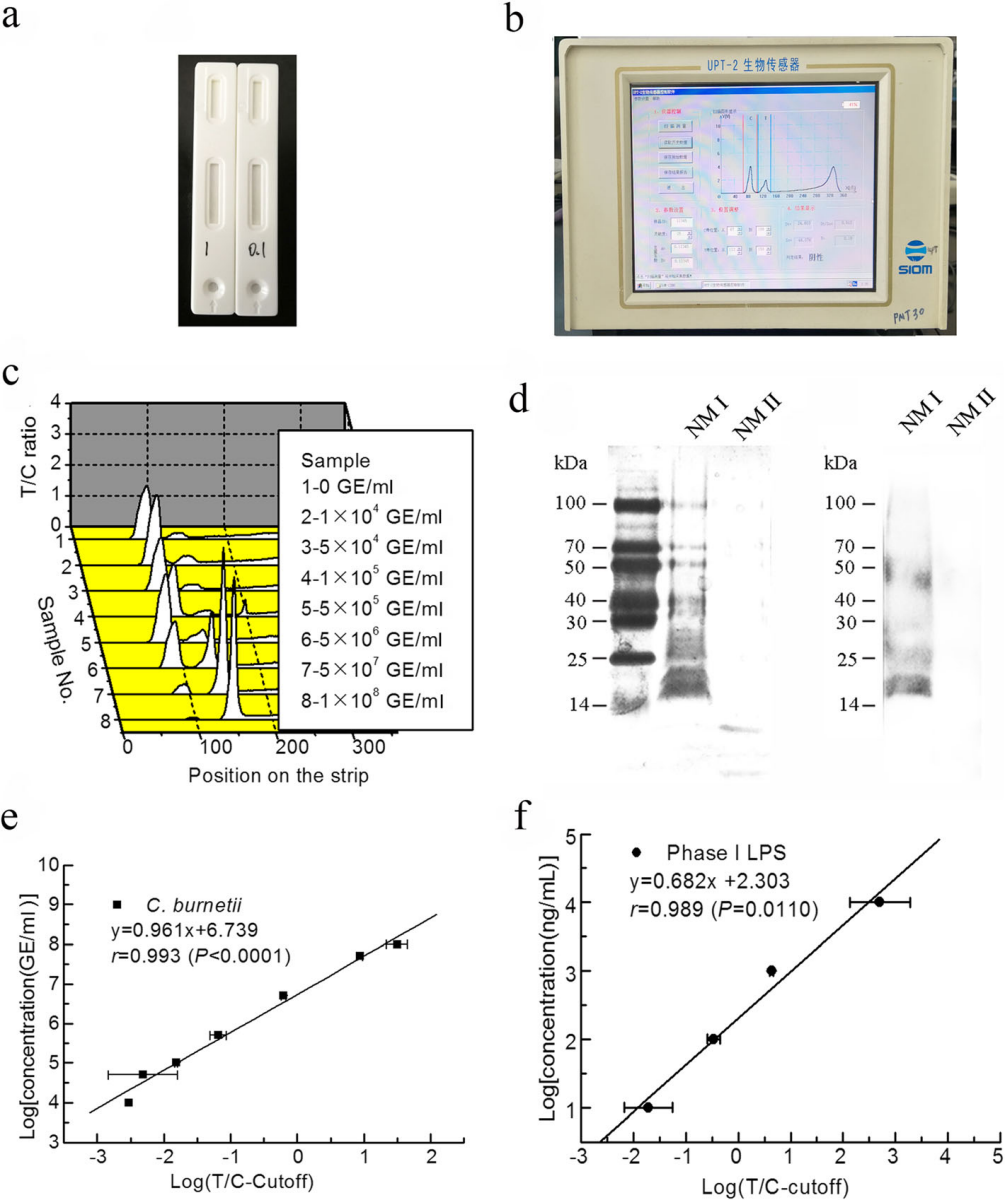
The detection limit, sensitivity, and precision of *Coxiella*-UPT-LF. **a** Photograph of a UPT-LF strip. **b** Photograph of the UPT biosensor. **c** Illustrations of the UPT assay for the detection of *C. burnetii* Xinqiao strain. Peaks on the left are signals for the control bands, and peaks on the right are for test bands. **d** LPS immunoblot of *C. burnetii* NMI and NMII. LPS of *C. burnetii* NMI and NMII was separated by SDS-PAGE, silver-stained, and probed with PI LPS-specific mAbs (10B5 and 10G7). **e** Standard curve for the quantification of *C. burnetii* Xinqiao strain by *Coxiella*-UPT-LF, with the logarithm of the difference between the T/C ratio and the cut-off value on the horizontal axis and the logarithm of the concentration (GE/ml) on the vertical axis. Data were expressed as mean ± SD, *n* = 3. **f** Standard curve for the quantification of NMI LPS by *Coxiella*-UPT-LF, with the logarithm of the difference between the T/C ratio and the cut-off value on the horizontal axis and the logarithm of the concentration (GE/ml) on the vertical axis. Data were expressed as mean ± SD, n = 3

The detection limit and sensitivity of *Coxiella*-UPT-LF for purified *C. burnetii* Xinqiao strain are 1 × 10^4^ and 5 × 10^4^ GE/ml, respectively. The detection limit and sensitivity of NMI LPS are 1 and 10 ng/ml, respectively. Coefficients of variation of *Coxiella*-UPT-LF for each concentration are defined as the ratios of standard deviations and means; they were all less than 15% for the detection of purified *C. burnetii* Xinqiao strain with concentrations below 5 × 10^7^ GE/ml and the detection of NMI LPS with concentrations below 1000 ng/ml. With increasing concentrations, the signals on the C band were too high for exact detection, which further influenced the precision of the strip. As expected, *Coxiella*-UPT-LF did not react with NMII or NMII LPS at any of the concentrations mentioned above. The specificity of mAbs to PI LPS was further confirmed by immunoblot with LPS extracted from different *C. burnetii* strains. As shown in Fig. 2d (also shown in Fig. S2 and Fig. S3), mAbs pooled from 10B5 and 10G7 reacted with NMI LPS, as indicated by a laddering profile above 14 kDa, but did not react with NMII LPS.

For linear quantitative correlation analysis, a standard curve was plotted for *Coxiella*-UPT-LF for purified *C. burnetii* Xinqiao strain from 1 × 10^4^ to 1 × 10^8^ GE/ml, as well as for NMI LPS from 10 to 10,000 ng/ml (Fig. 2e and f), with the logarithm of the difference between the T/C ratio and the cut-off value on the horizontal axis and the logarithm of the concentration on the vertical axis. The R values of the curves for purified *C. burnetii* Xinqiao and NMI LPS were both larger than 0.9, demonstrating the excellent accuracy for quantification.

### Specificity

**Fig. 3:**
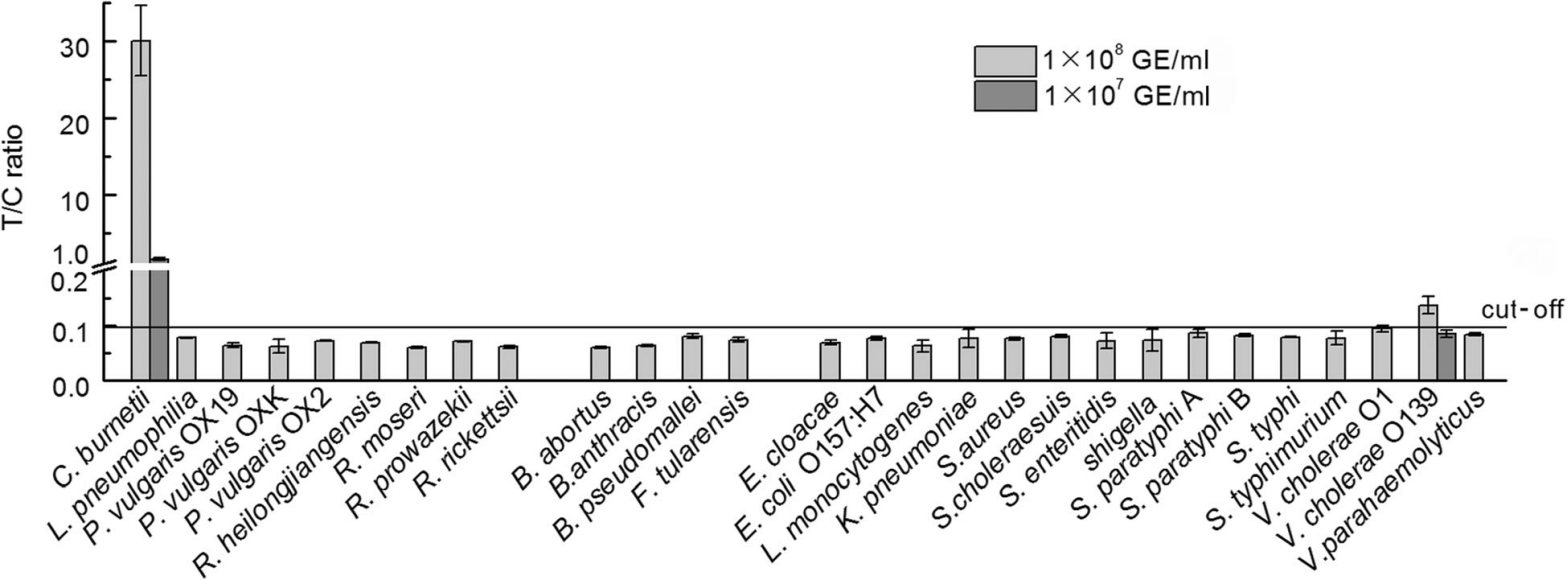
Evaluation of the specificity of *Coxiella*-UPT-LF. Except slight cross-reaction with *V. cholerae* O139, *Coxiella*-UPT-LF showed high specificity, without false-positive results for non-specific bacterial species. Data were expressed as mean ± SD, n = 3

Various purified bacteria with a concentration of 10^8^ GE/ml were used to evaluate the specificity of *Coxiella*-UPT-LF, including 8 genetically related bacteria, 4 bioterrorism agents, and 15 food-borne bacteria that have similar habitats as *C. burnetii*. As shown in Fig. 3, *Coxiella*-UPT-LF only exhibited slight cross-reaction with *Vibrio cholerae* O139 at a concentration of 10^8^ GE/ml. When *V. cholerae* O139 was added to the strip at a concentration of 10^7^ GE/ml, no cross-reaction was observed, indicating that *Coxiella*-UPT-LF has high specificity to *C. burnetii*.

### Inclusivity

**Fig. 4:**
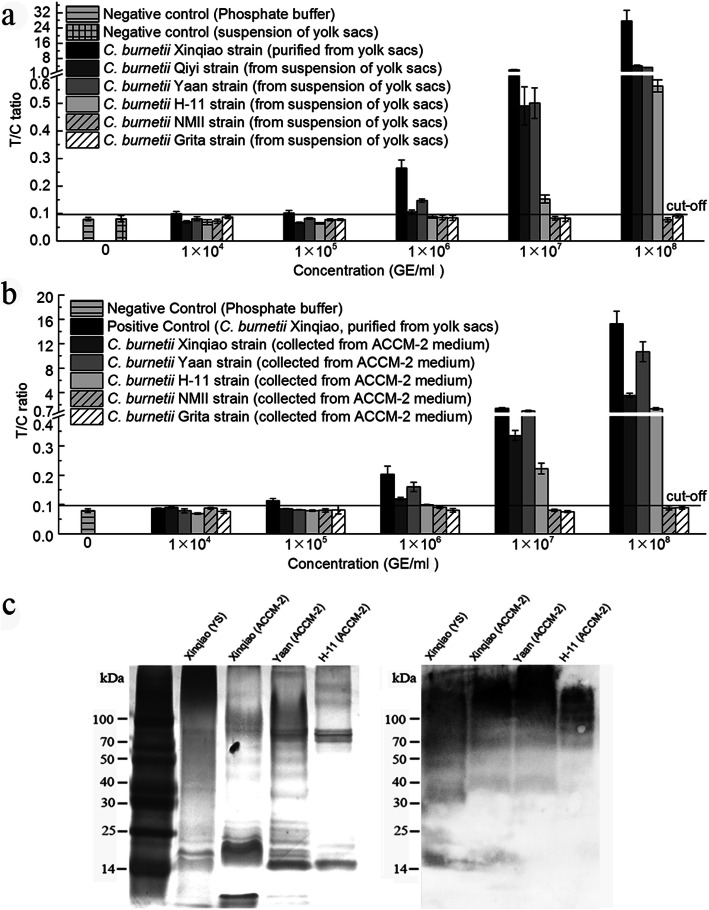
Inclusivity of *Coxiella*-UPT-LF for *C. burnetii*. **a** The inclusivity of *Coxiella*-UPT-LF for the detection of *C. burnetii* strains cultured in YS. Data were expressed as mean ± SD, *n* = 3. **b** The inclusivity of *Coxiella*-UPT-LF for the detection of *C. burnetii* strains cultured in ACCM-2 medium. Data were expressed as mean ± SD, n = 3. **c** LPS immunoblot of *C. burnetii* PI strains isolated in China. LPS of *C. burnetii* Xinqiao, Yaan, and H-11 strains was separated by SDS-PAGE, silver-stained, and probed with PI LPS-specific mAbs (10B5 and 10G7)

Five *C. burnetii* strains in YS suspension were used to assess the inclusivity of *Coxiella*-UPT-LF, i.e., three PI strains (*C. burnetii* Qiyi, Yaan, and H-11) and two PII strains (*C. burnetii* Grita and NMII). *C. burnetii* Xinqiao strain purified from YS served as a positive control. As negative controls, 10-, 100-, 1000-, and 10,000-fold dilutions of YS suspension and phosphate buffer were used. All of the negative controls had little influence on the detection of *Coxiella*-UPT-LF. As shown in Fig. 4a, the sensitivity of *Coxiella*-UPT-LF for *C. burnetii* Qiyi, Yaan, and H-11 in YS suspension was less than 1 × 10^6^, 1 × 10^6^, and 1 × 10^7^ GE/ml, respectively, and *Coxiella*-UPT-LF did not react with *C. burnetii* NMII and Grita, the strains of avirulent forms. The inclusivity of *Coxiella*-UPT-LF for PI *C. burnetii* and its non-reactivity with avirulent forms has practical significance for *C. burnetii* detection in the field.

To assess the influence of culture methods on *Coxiella*-UPT-LF, six *C. burnetii* strains, i.e., Xinqiao, Qiyi, Yaan, H-11, NMII, and Grita, were cultured in YS, transferred to acidified citrate cysteine medium-2 (ACCM-2), cultured for another five passages, and then detected by *Coxiella*-UPT-LF. However, the *C. burnetii* Qiyi strain grew poorly in ACCM-2 medium, as has previously been reported for other strains as well [13, 14]. The sensitivity of *Coxiella*-UPT-LF for *C. burnetii* Xinqiao, Yaan, and H-11 cultured in ACCM-2 was less than 1 × 10^6^, 1 × 10^6^, and 1 × 10^7^ GE/ml, respectively (Fig. 4b), which is slightly lower than or equal to that of bacteria in YS suspension.

The inclusivity of mAbs was further confirmed by immunoblotting with LPS of *C. burnetii* PI strains that were extracted from YS culture of *C. burnetii* Xinqiao and cell-free culture of Xinqiao, Yaan, and H-11. As shown in Fig. 4c (also shown in Fig. S4 and Fig. S5), mAbs pooled from 10B5 and 10G7 reacted with LPS of these strains, as indicated by a laddering profile above 14 kDa.

### Detection of *C. burnetii* from tissues of challenged mice and tick samples

**Fig. 5:**
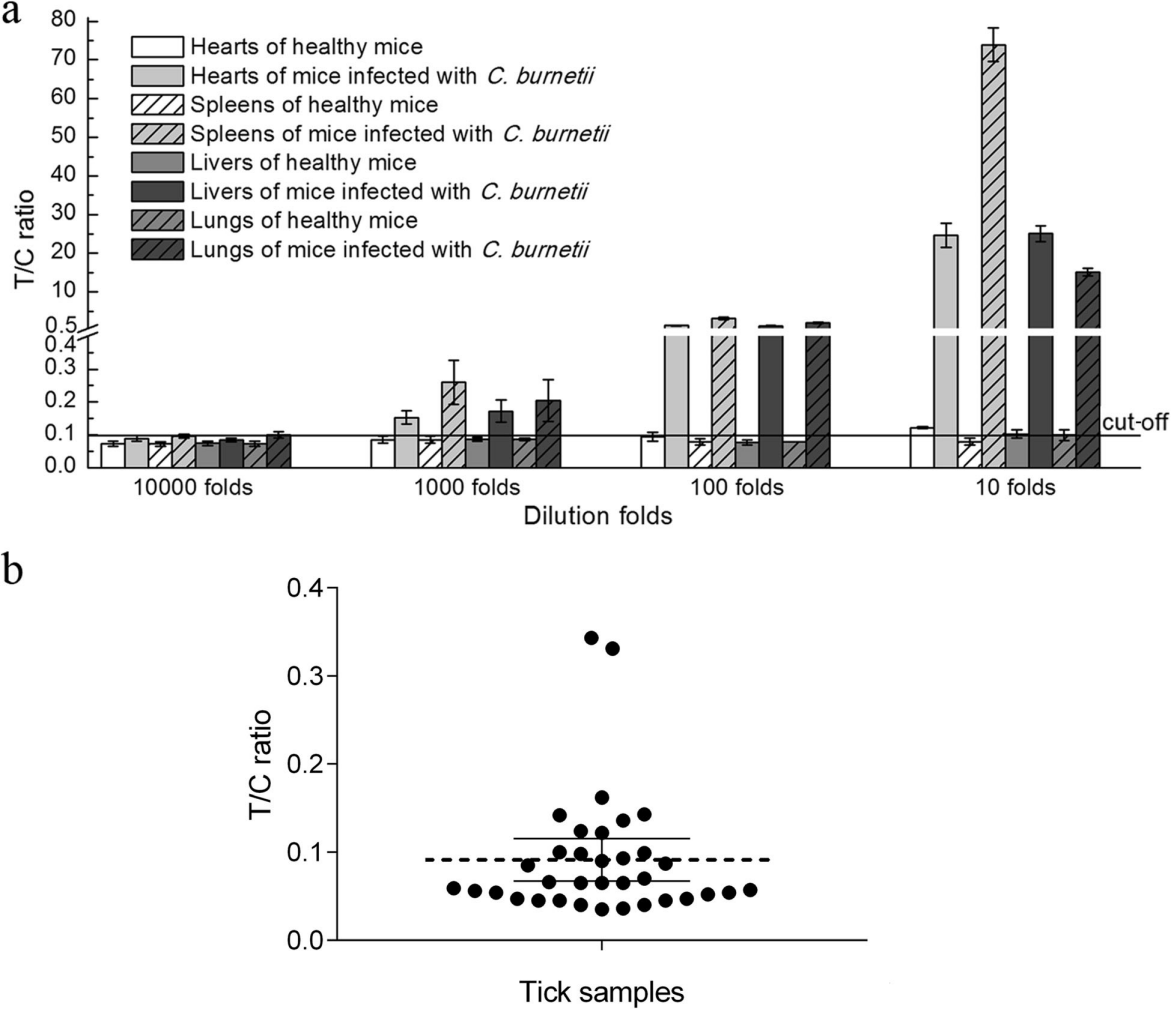
Detection results for *Coxiella*-UPT-LF of different *C. burnetii* strains in infected mice and ticks. **a** Detection results for *Coxiella*-UPT-LF of *C. burnetii* strains in organs from infected mice and control mice. Data were expressed as mean ± SD, n = 3. **b** Detection results for *Coxiella*-UPT-LF of *C. burnetii* strains in tick samples. Data were expressed as mean with 95% CI

To assess whether the *Coxiella*-UPT-LF assay can be used for the detection of infected samples, mice were challenged with 10^8^ GEs of *C. burnetii* Xinqiao strain purified from YS. On day 7 post-challenge, mice were killed, and tissues were analysed by *Coxiella*-UPT-LF assay. Tissues from healthy mice served as negative controls (Fig. 5a). For healthy mice, organ suspensions with 100- and 1000-fold dilutions have little influence on the *Coxiella*-UPT-LF assay, but T/C ratios of 10-fold dilutions of heart, liver, and lung suspensions were slightly higher than the cut-off value and were identified as false-positive results. For challenged mice, organ suspensions with 10-, 100-, and 1000-fold dilutions all tested positive, inferring that 1000-fold dilutions are suitable for *C. burnetii* detection. In addition, the numbers of *C. burnetii* gene copies in undiluted organ suspensions ranged from 1.7 × 10^8^ to 2.6 × 10^9^ GE/ml (Table S1), so the detection limits of the UPT-LF assay for *C. burnetii* in infected mouse samples may be more than 1 × 10^5^ GE/ml (the detection limit obtained in vitro). However, false positive results were observed when blood samples of both challenged and healthy mice were tested (Table S1). This was due to the signal reduction in the control line caused by antibodies in mouse blood that were irrelevant to *C. burnetii*. These antibodies reacted with the goat anti-mouse secondary antibodies on control line of *Coxiella*-UPT-LF which were intended to be combined with UCP-mAbs.

Moreover, ticks with sucked blood were collected from the field for evaluation of the *Coxiella*-UPT-LF assay. Thirty-five samples, with each sample containing about 228 ticks, were homogenised in PBS and analysed. PBS has little impact on *Coxiella*-UPT-LF, as its detection results are similar to those of non-saline phosphate buffer. As shown in Fig. 5b, 8 of 35 samples tested positive. The numbers of *C. burnetii* gene copies in these eight samples ranged from 2.4 × 10^5^ to 6.2 × 10^6^ GE/ml, as quantified by real-time PCR (Table S2), indicating that the detection limit of the UPT-LF assay for *C. burnetii* in naturally infected tick samples is higher than 2 × 10^5^ GE/ml.

## Discussion

Several mAbs against *C. burnetii* have previously been developed for diagnostic or vaccine use. A PI LPS targeting mAb and its variable fragments were developed for prophylaxis against *C. burnetii* exposure by Peng et al. [15]. A PI LPS targeting mAb was also developed by Palkovicova et al. for the detection of the virulent form of *C. burnetii* and for differentiation of the individual isolates [16, 17]. However, data on mAb-based assays for the quantitative detection of *C. burnetii* are rare. Therefore, in this study, we aimed to establish an mAb-based UPT-LF assay for the rapid and quantitative detection of *C. burnetii* PI strains, especially for use in the field.

In our *Coxiella*-UPT-LF assay, the detection limits were 10^4^ GE/ml for purified *C. burnetii* PI and 10 ng/ml for purified NMI LPS. Using 0.1 ml loading volume, the actual final amounts applied to the strip required for detection are 1000 *C. burnetii* bacteria and 1 ng of NMI LPS, indicating very high sensitivity for an immune-chromatography method used for rapid screening. The detection limits of *Coxiella*-UPT-LF for *C. burnetii* in YS suspension, in experimentally infected mice, and in naturally infected ticks were more than 10^5^ GE/ml, i.e., at least 10 times higher than that for the detection of purified *C. burnetii*. These results indicated that *Coxiella*-UPT-LF cannot fully distinguish *C. burnetii* from culture medium components or animal tissues.

*C. burnetii* Xinqiao, Yaan, Qiyi, and H-11 strains were isolated from China and characterised as PI strains [18]. *Coxiella*-UPT-LF recognised all these strains with different sensitivities, and additionally recognised LPS extracted from *C. burnetii* NMI, indicating that different isolates of *C. burnetii* PI strains may share the same LPS or other antigens, even though they differed in size and composition [3]. In addition, the *Coxiella*-UPT-LF assay showed good inclusivity for the detection of various isolates of *C. burnetii* PI strains, which might be of great importance when the assay is applied in the field, where no information on the isolates is available.

The sensitivity of the assay for the detection of Xinqiao strain purified from ACCM-2 medium was lower than that for the detection of the same strain purified from YS. In addition, PII-specific bands were observed in the LPS profiles of cell-free cultured Xinqiao strains. These results indicate that *C. burnetii* PI strains were undergoing phase transition when serially passaged in artificial medium [3, 14].

The physiology, lifestyle, and morphology of *Rickettsia* species are similar to those of *C. burnetii*, and *Legionella pneumonia* is closely related to *C. burnetii*, sharing genomic homology and causing similar clinical presentations. In addition to the above-mentioned bacteria, the conditioned pathogen *Proteus vulgaris*, which shares genomic homology with *C. burnetii*, bioterrorism agents, and food-borne bacteria that have similar habitats as *C. burnetii* must be distinguished from *C. burnetii* in the *Coxiella*-UPT-LF assay. The *Coxiella*-UPT-LF assay gave negative results for these bacterial strains, even at 10^8^ GE/ml, confirming the high specificity of the assay.

## Conclusions

Overall, the *Coxiella*-UPT-LF assay exhibited good sensitivity and specificity for the detection of *C*. *burnetii* PI strains in culture medium and in experimentally and naturally infected samples. Although further optimisation is necessary, *Coxiella*-UPT-LF is a reliable method for the rapid screening of *C. burnetii* and is suitable for on-site detection in the field.

## Methods

### Ethics

Thirteen female BALB/c mice were used for the preparation of mAb and infection with *C. burnetii*. This study complied with the Guidelines for the Welfare and Ethics of Laboratory Animals of China and was approved by the Committee of Welfare and Ethics of Laboratory Animals, Beijing Institute of Microbiology and Epidemiology (permit number: AMMS-2014-025).

### Bacteria, samples, and materials

All bacteria were preserved in our laboratory. *C*. *burnetii* PI strains that isolated in China, including Xinqiao, Qiyi, Yaan, and H-11 strain [18, 19], as well as *C*. *burnetii* PII strains, including NMII and Grita strain, were initially propagated in YS for 7–9 passages. Then the strains were transferred to ACCM-2 for another five passages in order to adapt to the cell-free culture system and to synchronise the passage numbers of the strains [20, 21]*.* Purified *C*. *burnetii* Xinqiao strain was separated from YS by renografin density centrifugation as previously described [22]. The concentrations of *C*. *burnetii* were determined by real-time PCR detecting the *com1* gene as previously described [23]. Purified LPS of NMI and NMII was provided by Dr. Chen Chen from the University of California, Berkeley as a generous gift. The LPS of other *C*. *burnetii* strains was extracted using a modified hot phenol method and analysed by SDS-PAGE as previously described [3].

*Legionella pneumophila* was cultured on BCYE agar plates. Bacterial suspensions of *P. vulgaris* OXK, *P. vulgaris* OX19, and *P. vulgaris* OX2 were purchased from Ningbo Tianrun Bio-Pharmaceutical Co. Ltd. (Ningbo, China). *Rickettsia moseri*, *Rickettsia prowazekii*, *Rickettsia rickettsii*, and *Rickettsia heilongjiangensis* were cultured in Vero cells or embryo cells and purified by differential centrifugation followed by centrifugation through 30% renografin [24]. Bioterrorism agents and the food-borne bacteria were all cultured in lysogeny broth, except for *Francisella tularensis*, *Brucella abortus*, and *Burkholderia pseudomallei*, which were cultured in brain heart infusion media. The concentrations of the bacteria were determined by real-time PCR.

UCP particles were purchased from Shanghai Kerune Phosphor Technology Co., Ltd. (Shanghai, China) and modified by our laboratory for combination with antibodies. Glass fibre and nitrocellulose membrane were purchased from Millipore Corp. (Bedford, MA, USA). Absorbent pad was purchased from Shanghai Goldbio Technology Co. Ltd. (Shanghai, China). The UPT biosensor was obtained from the Shanghai Institute of Optics and Fine Mechanics, the Chinese Academy of Sciences (Shanghai, China).

### mAb generation and sequence analysis

To generate mAbs against *C*. *burnetii*, five BALB/c mice were immunised with 20 μg of formalin-inactivated *C. burnetii* Xinqiao PI antigen four times at 3-week intervals. Then they were humanely sacrificed by cervical dislocation, and splenocytes were isolated. The positive hybridomas were obtained from the fusion of splenocytes from PI-immunised mice with SP2/0 myeloma cells and screened by ELISA for their ability to react with PI and PII antigens, as described previously [15]. Ascites were obtained by intraperitoneal injection of hybridomas into BALB/c mice, and antibodies in ascites were purified by caprylic acid/saturated ammonium sulphate precipitation. The antibody concentrations were determined by ultraviolet spectrophotometry, and their potencies were measured through serial two-fold dilutions. Finally, five antibodies were primarily selected: 10B5, 10G7, 13D6, 6D8, and 8A1.

To determine the cDNA-derived amino acid sequences of the VH and VL chains of these mAbs, total RNA was extracted from 10^6^ mAb producing hybridoma cells using the Qiagen RNeasy Mini Kit (Qiagen, Valencia, CA). The cDNA of hybridoma cells was synthesised by the 5′-RACE method. The genes of VH and VL were amplified from the synthesised cDNA by PCR and sequenced by Genewiz Co. Ltd. (Suzhou, China). The sequences were compared with the mouse IgG database using IMGT/V-Quest (www.imgt.cines.fr/home.html). The automatic modelling of mAb variable domains was established by the canonical structure method from abYmod (http://abymod.abysis.org/).

### Establishment of the UPT assay

The mAbs (2 mg/ml) against *C. burnetii* and goat anti-mouse were dispensed at a speed of 1 μl/cm as test line (T) and control line (C) on nitrocellulose membranes. UCPs were conjugated with different antibodies to prepare different conjugation pads. The nitrocellulose membrane with each antibody was combined with various conjugation pads, attached to a sticky base with absorbent papers and sample pads, and finally cut into 4 mm pieces to fabricate different UPT strips.

The primary sample treating buffer (0.03 M phosphate buffer containing 0.5% IGEPAL CA-630) was used for the selection of antibodies suitable for UPT detection and mixed with *C. burnetii* Xinqiao strain purified from YS at serial concentrations (10^7^, 10^8^, and 10^9^ GE/ml) at a ratio of 9:1 before loading to each strip. As a negative control, 0.03 M phosphate buffer was used. After 15 min, the strip was put into the UPT biosensor to read the signal intensities of the T and C bands. The T/C ratios were used as the detection results. The results of *C. burnetii* and phosphate buffer samples were abbreviated as P and N. To evaluate the performance of each UPT-LF strip (fabricated with different antibodies), the (P − N)/N ratio was calculated. The UPT-LF strips with the highest (P − N)/N ratios were selected for subsequent experiments.

The method for labelling UCPs and antibodies and the components of sample treating buffer were further optimised. The final optimised sample treating buffer was dispensed on the conjugation pad and dried at 37 °C for 1 h. Samples could be directly applied on the strips.

### Evaluation of detection limit, sensitivity, and precision

To determine the cut-off value of *Coxiella*-UPT-LF, 0.1 ml of 0.03 M phosphate buffer was applied to the strip. Phosphate buffer was also used as a negative control. *C. burnetii* Xinqiao strain purified from YS was diluted in 0.03 M phosphate buffer into 1 × 10^3^, 5 × 10^3^, 1 × 10^4^, 5 × 10^4^, 1 × 10^5^, 5 × 10^5^, 5 × 10^6^, 5 × 10^7^, and 1 × 10^8^ GE/ml, while LPS from NMI and NMII was diluted to 1, 10, 100, 1000, and 10,000 ng/ml for UPT detection. Each dilution was added to three *Coxiella*-UPT-LF strips.

For LPS immunoblotting, *C. burnetii* LPS was extracted, transferred to a PVDF membrane, and incubated with pooled 10G7 and 10B5 mAbs at 1:10,000 dilution and then HRP-conjugated goat anti-mouse IgM. LPS was detected by chemiluminescence as previously described [3].

### Evaluation of specificity

Numerous bacteria at a concentration of 10^8^ GE/ml were used to evaluate the specificity of *Coxiella*-UPT-LF. Genetically related bacteria included *L. pneumophila*, *P. vulgaris* OXK, *P. vulgaris* OX19, *P. vulgaris* OX2, *R. heilongjiangensis*, *R. moseri*, *R. prowazekii*, and *R. rickettsii.* Bioterrorism agents included *B. abortus*, *Bacillus anthracis*, *B. pseudomallei*, and *F. tularensis*. The food-borne pathogens with similar habitats to *C. burnetii* included *Enterobacter cloacae*, *Escherichia coli* O157, *Listeria monocytogenes*, *Klebsiella pneumoniae*, *Staphylococcus aureus*, *Salmonella enterica* serotype *choleraesuis*, *Salmonella enteritidis*, *Shigella*, *Salmonella paratyphi* A, *S. paratyphi* B, *Salmonella typhi*, *Salmonella typhimurium*, *V. cholerae* O1, *V. cholerae* O139, and *Vibrio parahaemolyticus*. The bacterial samples were diluted in 0.03 M phosphate buffer and directly applied to the *Coxiella*-UPT-LF assay. Each sample was tested in triplicate.

### Evaluation of inclusivity

To assess the capacity of *Coxiella*-UPT-LF to detect diverse *C. burnetii* strains, three PI strains of *C. burnetii* were employed, i.e., *C. burnetii* Qiyi, Yaan, and H-11, in YS suspension. To evaluate the influences of culture methods on the detection, *C. burnetii* strains cultured in ACCM-2 medium were collected through centrifugation. All of the bacterial samples were diluted to final concentrations of 1 × 10^4^, 1 × 10^5^, 1 × 10^6^, 1 × 10^7^, and 1 × 10^8^ GE/ml in 0.03 M phosphate buffer. The concentrations were confirmed by real-time PCR detection of the *com*1 gene of *C. burnetii* [23]. Then, 0.1 ml of the prepared samples was applied to *Coxiella*-UPT-LF strips. Suspension of normal yolk sac was also diluted 10-, 100-, 1000-, and 10,000-fold to evaluate its influence on the detection of *Coxiella*-UPT-LF. Each sample was tested in triplicate.

### Application of the *Coxiella*-UPT-LF assay to experimentally and naturally infected samples

Tissues of mice challenged with *C. burnetii* were used as infected samples for the evaluation of *Coxiella*-UPT-LF. Eight female, 6-week-old BALB/c mice were randomly divided into two groups. In the infected group, five mice were infected with 10^8^ GE *C. burnetii* Xinqiao strain through intraperitoneal injection. In the control group, three mice were injected with PBS. One week after infection, mice were humanely sacrificed by cervical dislocation, and their spleens, lungs, livers, hearts and blood were collected. Then the organs were homogenised into cell suspensions and the whole blood was lysed into suspensions. The suspensions were diluted 10-, 100-, and 1000-fold in 0.03 M phosphate buffer for sample preparation, and then 0.1 ml was directly applied to the strip. The DNA from organ suspensions was extracted using the DNeasy Blood & Tissue Kit (QIAGEN), and the bacterial burden was quantified by real-time PCR as described above.

Eight thousand ticks with sucked blood were collected from the forest steppe field in public places of Tianshan Mountain, Wusu City, Xinjiang province, China. They were divided into 35 portions. Each portion, with about 228 ticks, was ground into powder and vortexed into suspension in PBS. The suspensions were deposited for 2–4 min, and the supernatants were directly applied in *Coxiella*-UPT-LF, with PBS as a negative control. The DNA of each sample was extracted using the DNeasy Blood & Tissue Kit (QIAGEN), and the bacterial burden was quantified by real-time PCR as described above.

## Supplementary information

**Additional file 1: Figure S1.** Amino acid sequence and models of variable domains of 10B5 and 10G7. (a) Analysis of amino acid sequence of VL chains of 10B5 and 10G7; (b) Analysis of amino acid sequence of VH chains of 10B5 and 10G7; (c) Models of variable domains of 10B5 and 10G7 established by the molecular modeling.

**Additional file 2: Figure S2.** Raw image of LPS profile of *C. burnetii* NMI and NMII determined by silver stain.

**Additional file 3: Figure S3.** Raw image of LPS profile of *C. burnetii* NMI and NMII determined by immunoblot.

**Additional file 4: Figure S4.** Raw image of LPS profile of *C. burnetii* PI strains isolated in China determined by silver stain.

**Additional file 5: Figure S5.** Raw image of LPS profile of *C. burnetii* PI strains isolated in China determined by immunoblot.

**Additional file 6: Table S1.** The T/C ratios of UPT-LF and the *C. burnetii* gene copies for organ suspensions of infected mice.

**Additional file 7: Table S2.** The T/C ratios of UPT-LF and the *C. burnetii* gene copies for tick samples.

## Data Availability

All data generated or analyzed during current study are available from the corresponding author on reasonable request.

## Abbreviations

ACCM-2: Acidified citrate cysteine medium-2
*C. burnetii*: *Coxiella burnetii*
IFA: Immunofluorescence assay
LPS: Lipopolysaccharide
NMI: Nine Mile phase I strain
NMII: Nine Mile phase II strain
PI: Phase I
PII: Phase II
POCT: Point-of-care testing
UCPs: Up-converting phosphor particles
UPT-LF: Up-converting phosphor technology-based lateral flow
YS: Yolk sacs
VH: Variable heavy
VL: Variable light
