# Supplementary figures and images for: Development and evaluation of an up-converting phosphor technology-based lateral flow assay for rapid and quantitative detection of Coxiella burnetii phase I strains
Source: BMC Microbiol. 2020 Aug 12;20:251. doi: 10.1186/s12866-020-01934-0 (PMC7425161; doi:10.1186/s12866-020-01934-0)

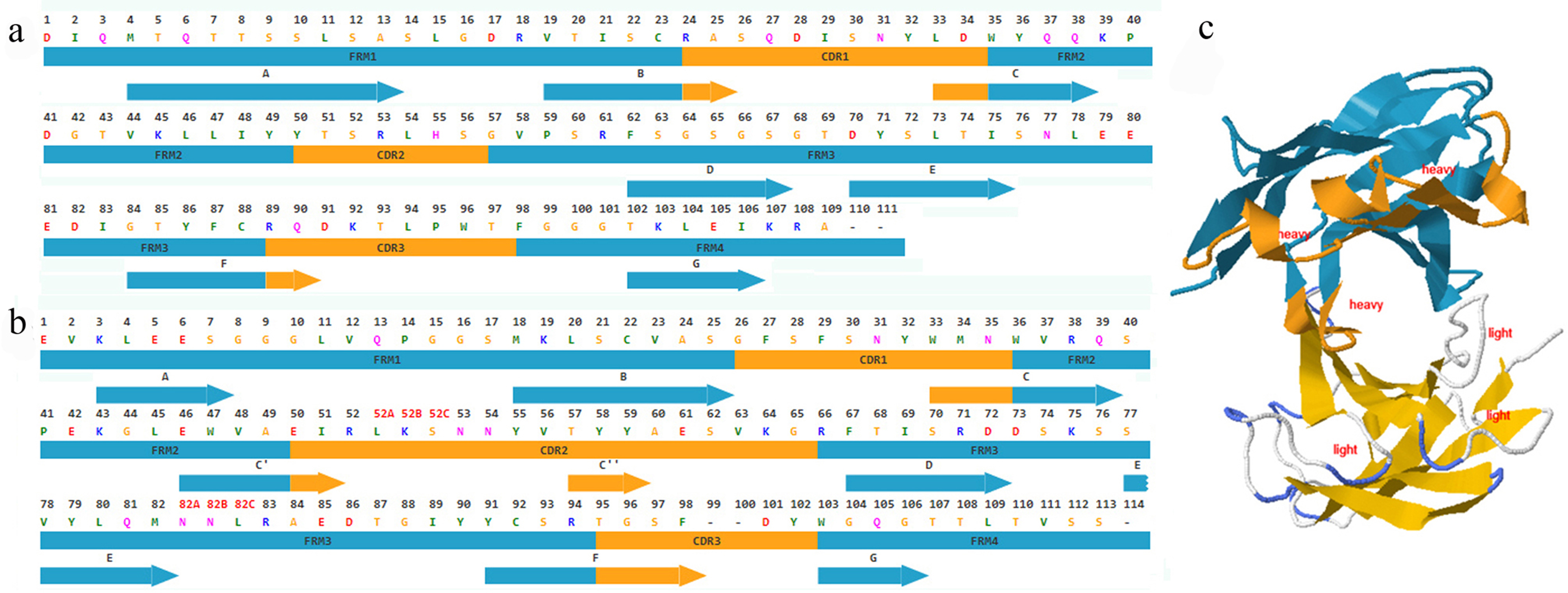

Supplement: Supplementary file 1 — Additional file 1: Figure S1. Amino acid sequence and models of variable domains of 10B5 and 10G7. (a) Analysis of amino acid sequence of VL chains of 10B5 and 10G7; (b) Analysis of amino acid sequence of VH chains of 10B5 and 10G7; (c) Models of variable domains of 10B5 and 10G7 established by the molecular modeling. [file 12866_2020_1934_MOESM1_ESM.tif]

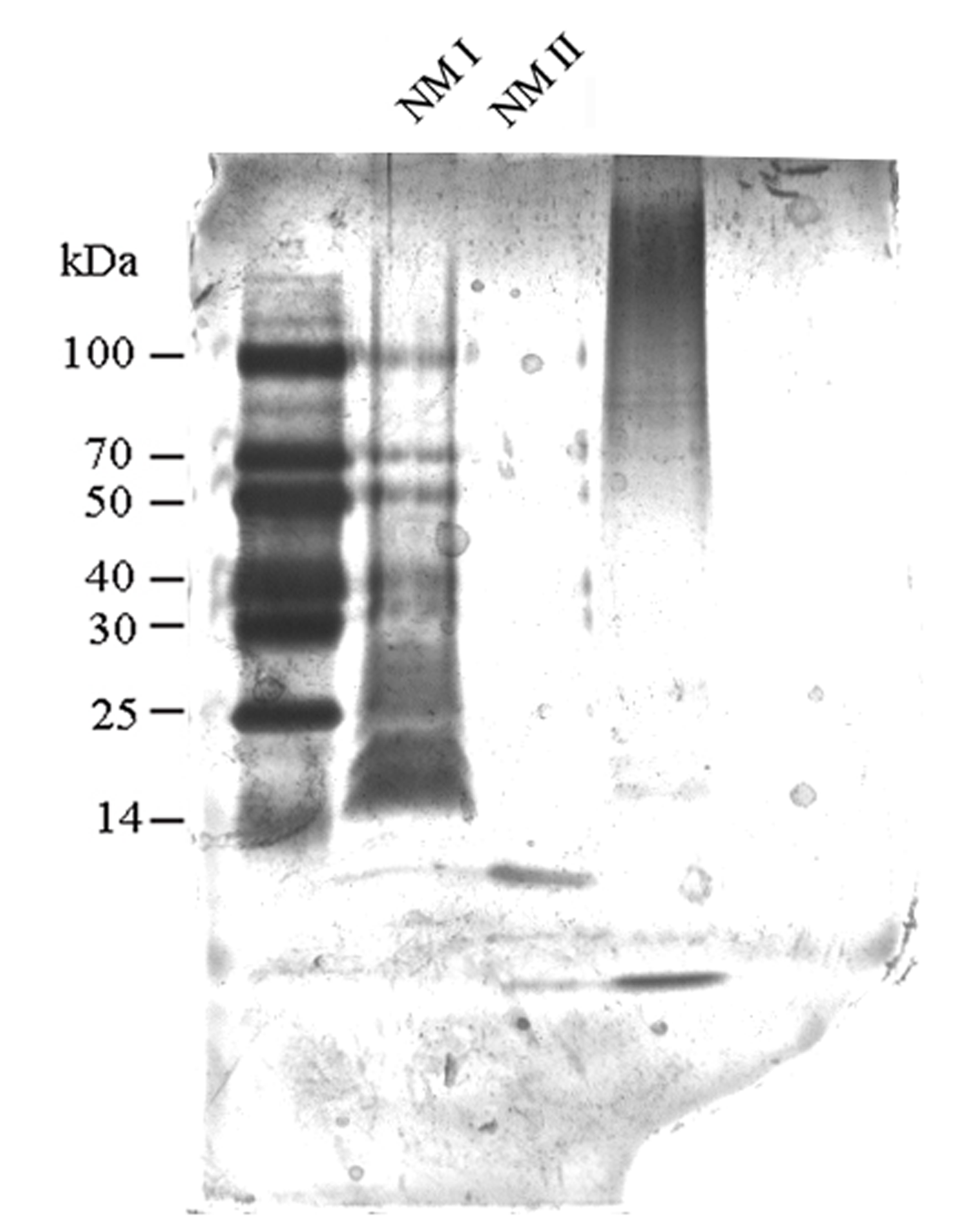

Supplement: Supplementary file 2 — Additional file 2: Figure S2. Raw image of LPS profile of C. burnetii NMI and NMII determined by silver stain. [file 12866_2020_1934_MOESM2_ESM.tif]

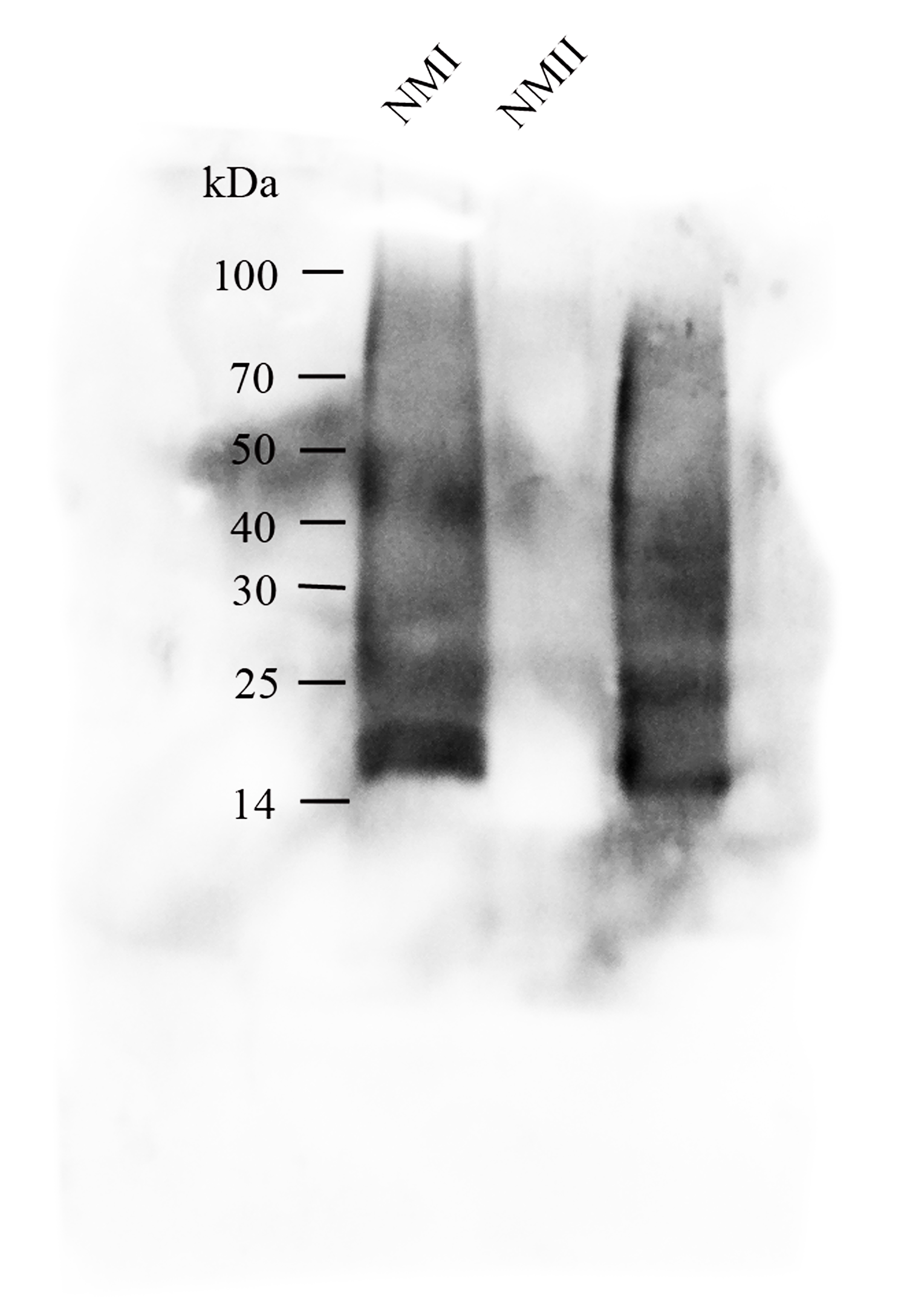

Supplement: Supplementary file 3 — Additional file 3: Figure S3. Raw image of LPS profile of C. burnetii NMI and NMII determined by immunoblot. [file 12866_2020_1934_MOESM3_ESM.tif]

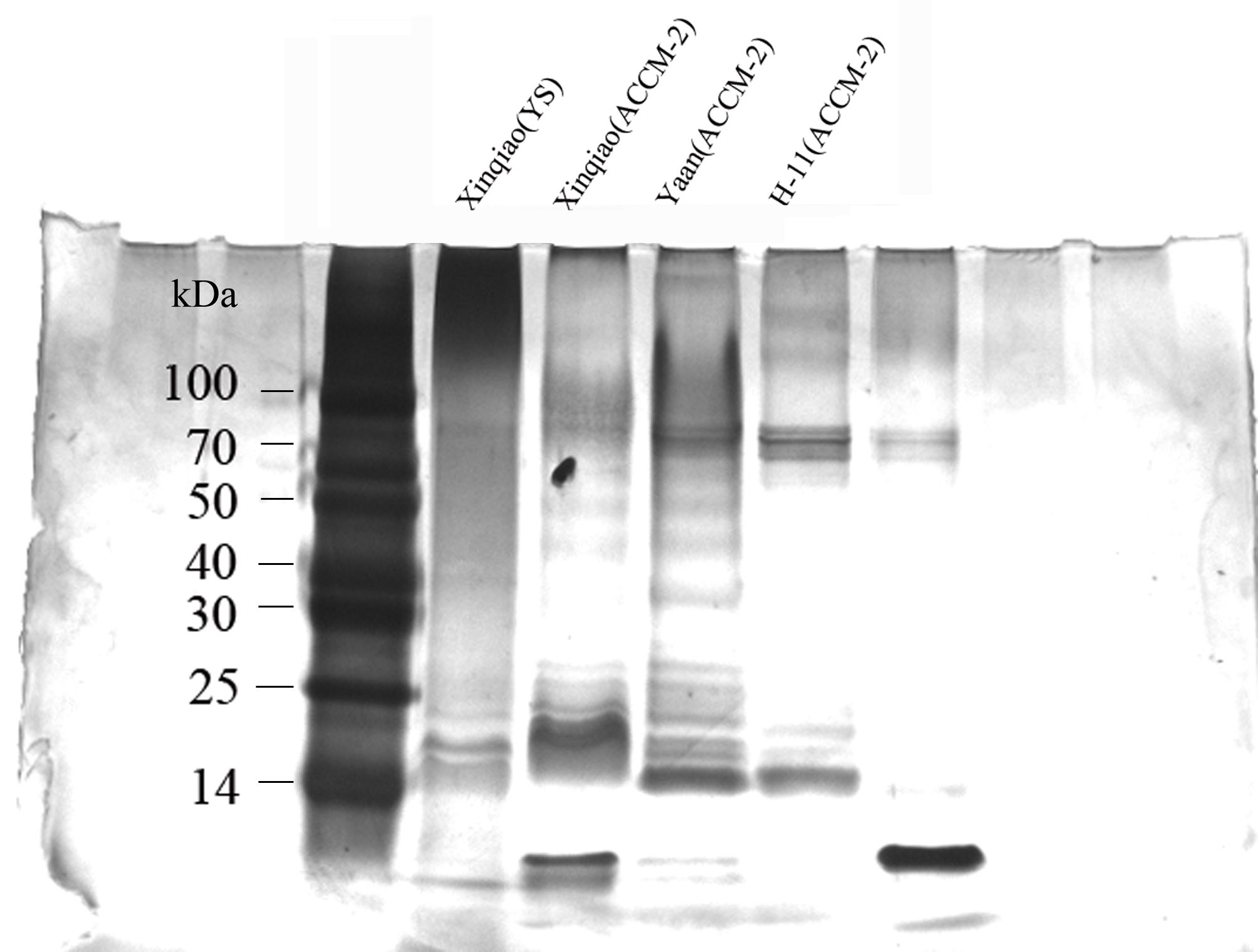

Supplement: Supplementary file 4 — Additional file 4: Figure S4. Raw image of LPS profile of C. burnetii PI strains isolated in China determined by silver stain. [file 12866_2020_1934_MOESM4_ESM.tif]

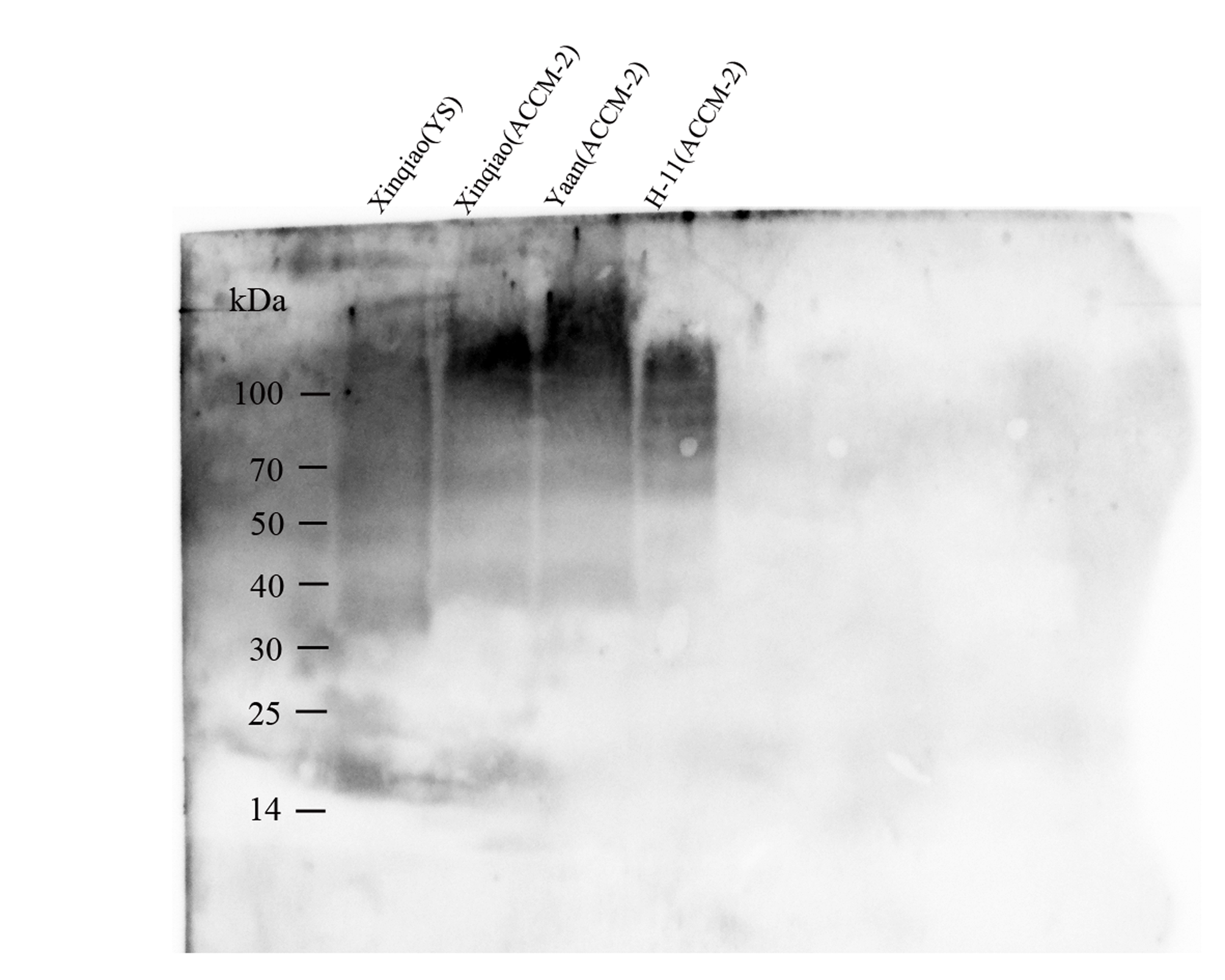

Supplement: Supplementary file 5 — Additional file 5: Figure S5. Raw image of LPS profile of C. burnetii PI strains isolated in China determined by immunoblot. [file 12866_2020_1934_MOESM5_ESM.tif]
